# Intraoperative neuromonitoring during thyroidectomy does not decrease vocal cord palsy risk, but the cumulative experience of the surgeon may

**DOI:** 10.1007/s00595-024-02871-5

**Published:** 2024-06-06

**Authors:** Hye Lim Bae, Moon Young Oh, Mira Han, Che-Wei Wu, Young Jun Chai

**Affiliations:** 1 Department of Surgery, Seoul National University College of Medicine, Seoul National University Hospital, Seoul, Korea; 2 Department of Surgery, Seoul National University College of Medicine, Seoul Metropolitan Government Seoul National University Boramae Medical Center, Seoul, Korea; 3 Medical Research Collaborating Center, Seoul Metropolitan Government Seoul National University Boramae Medical Center, Seoul, Korea; 4 Department of Otorhinolaryngology—Head and Neck Surgery, Kaohsiung Medical University Hospital, Kaohsiung, Taiwan; 5 Transdisciplinary Department of Medicine and Advanced Technology, Seoul National University Hospital, Seoul, Korea

**Keywords:** Intraoperative neuromonitoring, Risk factors, Thyroid cancer, Thyroidectomy, Vocal cord palsy

## Abstract

**Purpose:**

To evaluate the influence of intraoperative neuromonitoring (IONM) on vocal cord palsy (VCP) rates and assess the temporal trends in VCP rates.

**Methods:**

The subjects of this retrospective study were patients who underwent thyroidectomy for thyroid cancer between March, 2014 and June, 2022, at a university hospital in Korea. We compared VCP rates between the non-IONM and IONM groups and analyzed the risk factors for VCP and VCP rates over time.

**Results:**

A total of 712 patients were included in the analysis. The rates of transient and permanent VCP did not differ significantly between the non-IONM and IONM groups. Transient VCP occurred in 4.6% and 4.3% patients (*p* = 0.878) and VCP was permanent in 0.7% and 0.4% patients (*p* = 0.607) in the non-IONM and IONM groups, respectively. Among the nerves at risk, transient damage occurred in 2.8% and 3.0% patients (*p* = 0.901), and permanent damage occurred in 0.4% and 0.3% (*p* = 0.688), respectively. Multivariate analysis revealed no significant risk factors for VCP. There was a significant decreasing trend in VCP rates over time as the cumulative number of cases increased (*p* = 0.017).

**Conclusions:**

IONM did not reduce the risk of VCP significantly. However, the declining trend of VCP rates suggests that the surgeon’s experience may mitigate VCP risk.

## Introduction

The recurrent laryngeal nerve (RLN) branches off the vagus nerve (VN) and follows a course adjacent to the medial side of the thyroid along the tracheoesophageal groove [1]. RLN injury is a major complication associated with thyroidectomy [2, 3]. RLN injury can lead to impaired movement of the vocal cords, known as vocal cord palsy (VCP), causing symptoms, such as hoarseness, increased risk of aspiration, and breathing difficulties [4].

To preserve the RLN during thyroidectomy, various techniques and devices have been introduced, including intermittent intraoperative nerve monitoring (IONM) [5, 6]. IONM involves stimulating either the RLN or VN during thyroidectomy, converting vocal cord muscle movement into an electronic response and recording the evoked electromyographic signal, thereby aiding in the identifying the nerve [7]. However, there is controversy about whether IONM effectively reduces VCP rates. While some studies suggest that IONM can lower the incidence of VCP and predict the extent of nerve damage after thyroidectomy [8, 9], others report no significant difference in VCP rates between patients who undergo IONM and those who do not [10, 11] Moreover, the retrospective nature of these studies makes them susceptible to selection bias, as IONM is used more frequently in patients at higher risk of RLN injury [12].

The introduction and adoption of IONM vary among healthcare organizations and regions [13]. Inconsistencies in the availability and cost of IONM are attributed to differences in healthcare systems and health insurance coverage [14]. In Korea, the utilization of IONM was previously limited by a lack of national health insurance coverage; however, since December, 2016, IONM has been covered by national health insurance, leading to rapid expansion of its usage. This allowed us to conduct a study in which patients were assigned to surgical groups (non-IONM vs. IONM) based on their date of surgery rather than the surgeon’s preference or patient-related factors, thereby reducing selection bias. The objective of this study was to evaluate the effectiveness of IONM in preventing VCP and to identify the factors associated with VCP in patients undergoing thyroidectomy, stratified by the date of surgery.

## Materials and methods

### Study population

A retrospective medical record analysis was conducted on consecutive patients diagnosed with thyroid carcinoma who underwent thyroidectomy at Seoul Metropolitan Government–Seoul National University Boramae Medical Center between March, 2014 and June, 2022. All patients underwent preoperative ultrasonography and computed tomography of the neck to assess the tumor extent and lymph node status. The routine use of IONM for patients undergoing thyroidectomy at our center began in December, 2016, which coincides with the national health insurance coverage for IONM. Thus, patients who underwent thyroidectomy between March, 2014 and November, 2016 were included in the non-IONM group and those who underwent thyroidectomy between December, 2016 and June, 2022 were included in the IONM group. Patients with a previous history of neck surgery, simultaneous parathyroidectomy or thymectomy, preoperative RLN nerve invasion, or tracheal tumor invasion were excluded from the analysis. The study was approved by the Institutional Review Board of Seoul Metropolitan Government–Seoul National University Boramae Medical Center (IRB number: 10–2023-30), and individual consent for this retrospective analysis was waived.

### Surgical and intraoperative neuromonitoring procedures

On induction of anesthesia, patients received lidocaine (30 mg), rocuronium (0.6 mg/kg), and a targeted-controlled continuous infusion of propofol and remifentanil. All operations were performed by a single surgeon (Y.J.C). For patients who underwent thyroidectomy without IONM, the RLNs were identified visually before and after thyroidectomy. In those who underwent thyroidectomy with IONM, standard reinforced electromyogram endotracheal tubes and the nerve integrity monitor 3.0 system (Medtronic, Jacksonville, FL, USA) were utilized, and the IONM procedure followed the standardized guidelines of the International Neural Monitoring Study Group [15]. Loss of signal was defined as an RLN amplitude level lower than 100 μV during surgery.

### Postoperative evaluation

Postoperative assessments were performed during outpatient clinic appointments and vocal cords were evaluated by laryngoscopy within 10 days after thyroidectomy. VCP was defined as fixed or hypomobile vocal cords observed on indirect laryngoscopy. If VCP was identified, further follow-up was conducted regularly until the palsy improved. RLN injury was categorized as ‘transient’ if movement of the vocal cords recovered within 6 months of surgery and ‘permanent’ if impairment persisted for over 6 months. Serum levels of total calcium and parathyroid hormone were monitored to identify postoperative hypoparathyroidism. Hypoparathyroidism was defined by laboratory findings of parathyroid hormone levels below 15.0 pg/ml or the need for an oral calcium preparation to treat symptoms, such as numbness or tingling sensation in the hands, feet, or face.

### Clinicopathological characteristics and surgical outcomes

We evaluated the clinicopathological characteristics of the patients and compared the surgical outcomes of the IONM and non-IONM groups. Patient characteristics included age, gender, and body mass index (BMI), and surgical characteristics included surgical extent and extent of lymph node dissection. Histopathological characteristics included lesion location and size, multicentricity, extrathyroidal extension, histological type, number of retrieved lymph nodes, number of metastatic lymph nodes, and the presence of thyroiditis. Cancer stage was classified using the American Joint Committee on Cancer staging manual 8th edition [16]. The mechanism of injury of each case of VCP was based on the operative records and categorized into five types: traction injury, crush injury, transection injury, thermal injury, and undetermined [17, 18]. VCP was further classified as caused by either RLN or VN injury.

### Statistical analysis

Patient characteristics are presented as frequencies with percentages for categorical variables and as the mean ± standard deviation or median with interquartile range for continuous variables. Continuous variables were compared using the *t* test or Mann–Whitney *U* test, whereas categorical variables were compared using the Chi-square or Fisher’s exact tests. Risk factors associated with VCP were examined using univariate logistic regression models and then considered for inclusion in the multivariable logistic regression model if significant (*p* value < 0.1). The trend of the VCP rate over time was analyzed using the Cochran–Armitage trend test. Statistical analyses were performed using SAS version 9.4 (SAS Institute Inc., Cary, NC) and R version 4.2.2 (R foundation for Statistical Computing, Vienna, Austria). Except for the inclusion criteria of *p* less than 0.1 used for the multivariate logistic regression model, *p* values less than 0.05 were considered significant.

## Results

### Clinical characteristics

**Fig. 1:**
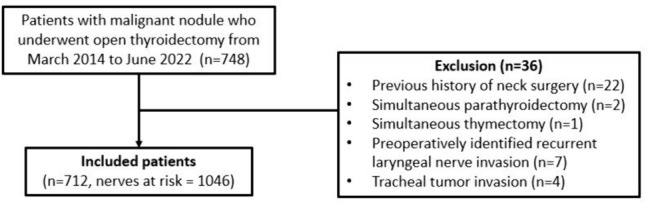
Flow diagram of included patients. After exclusion, 712 patients (151 non-IONM and 561 IONM) and 1046 nerves at risk (246 non-IONM and 800 IONM) were included in the study. *IONM* intraoperative neuromonitoring

A total of 748 patients underwent thyroidectomy between March, 2014 and June, 2022. After the exclusion of 36 patients, 712 patients (485 female and 227 male) were included in the analysis (Fig. 1). Table 1 summarizes the clinicopathological characteristics of the included patients. The mean age was 52.6 years and the mean BMI was 25.3 kg/m^2^. The non-IONM group comprised 151 patients and the IONM group comprised 561 patients. There were no significant differences in patient characteristics between the groups, including sex, age, and BMI. Lobectomy and total thyroidectomy were performed in 56 (37.1%) and 95 (62.9%) patients, respectively, in the non-IONM group, while 322 (57.4%) and 239 (42.6%) patients underwent lobectomy and total thyroidectomy, respectively, in the IONM group (*p* < 0.001). The non-IONM group had a higher proportion of central node dissection than the IONM group (98.0% vs. 93.2%, *p* = 0.025). The non-IONM group was associated with lower T stages, whereas the IONM group was associated with higher T stages (*p* < 0.001). There were no differences in other surgical or histopathological characteristics between the groups. Final pathology included 688 (93.8%) papillary thyroid carcinomas, 31 (4.4%) follicular thyroid carcinomas, 5 (0.7%) oncocytic carcinomas, 4 (0.6%) medullary thyroid carcinomas, 2 (0.3%) poorly differentiated thyroid carcinomas, and 2 (0.3%) anaplastic carcinomas.

**Table 1:** Clinicopathological characteristics

Variables	Total (*n* = 712)	Non-IONM (*n* = 151)	IONM (*n* = 561)	*p* value
*Sex*				
Female	485 (68.1)	108 (71.5)	377 (67.2)	0.312^1^
Male	227 (31.9)	43 (28.5)	184 (32.8)	
Age (years)	52.6 ± 13.1	51.8 ± 13.9	52.8 ± 12.9	0.408^3^
Body mass index (kg/m^2^) (N = 707)	25.3 ± 4.0	25.0 ± 3.4	25.4 ± 4.2	0.237^3^
*Tumor location*				
Right	307 (43.1)	66 (43.7)	241 (43.0)	0.499^1^
Left	274 (38.5)	62 (41.1)	212 (37.8)	
Bilateral	131 (18.4)	23 (15.2)	108 (19.3)	
*Surgical extent*				
Lobectomy	378 (53.1)	56 (37.1)	322 (57.4)	< .0001^1^
Total thyroidectomy	334 (46.9)	95 (62.9)	239 (42.6)	
*Central node dissection*				
Not Done	41 (5.8)	3 (2.0)	38 (6.8)	0.025^1^
Done	671 (94.2)	148 (98.0)	523 (93.2)	
*Lateral node dissection*				
Not Done	625 (87.8)	137 (90.7)	488 (87.0)	0.213^1^
Done	87 (12.2)	14 (9.3)	73 (13.0)	
*Thyroid cancer pathology*				
Papillary thyroid carcinoma	688 (93.8)	146 (96.7)	522 (93.0)	0.253^2^
Follicular thyroid carcinoma	31 (4.4)	3 (2.0)	28 (5.0)	
Oncocytic carcinoma	5 (0.7)	0 (0.0)	5 (0.9)	
Medullary thyroid carcinoma	4 (0.6)	2 (1.3)	2 (0.4)	
Poorly differentiated thyroid carcinoma	2 (0.3)	0 (0.0)	2 (0.4)	
Anaplastic thyroid carcinoma	2 (0.3)	0 (0.0)	2 (0.4)	
*T stage*				
T1	580 (81.5)	134 (88.7)	446 (79.5)	0.001^1^
T2	67 (9.4)	15 (9.9)	52 (9.3)	
T3 or more	65 (9.1)	2 (1.3)	63 (11.2)	
*N stage*				
N0	420 (59.0)	87 (57.6)	333 (59.4)	0.214^1^
N1a	203 (28.5)	50 (33.1)	153 (27.3)	
N1b	89 (12.5)	14 (9.3)	75 (13.4)	
Harvested lymph nodes (*n*)	8.4 ± 11.2	5.0 (2.0–9.0)	5.0 (2.0–10.0)	0.631^4^
*Multifocality*				
Single	494 (69.4)	104 (68.9)	390 (69.5)	0.879^1^
Multifocal	218 (30.6)	47 (31.1)	171 (30.5)	
*Extrathyroidal extension*				
None	656 (92.1)	139 (92.1)	517 (92.2)	0.966^1^
Gross	56 (7.9)	12 (7.9)	44 (7.8)	
*Thyroiditis*				
No	524 (73.6)	111 (73.5)	413 (73.6)	0.979^1^
Yes	188 (26.4)	40 (26.5)	148 (26.4)	

Values are expressed as *n* (%) or mean ± standard deviation

^1^Chi-square test

^2^Fisher’s exact test

^3^* T* test

^4^Wilcoxon rank sum test

*IONM* Intraoperative nerve monitoring, *CND* Central node dissection, *LND* Lateral node dissection

### Postoperative surgical complications

Table 2 lists the postoperative complications in the non-IONM and IONM groups. There were no significant differences between the groups. Transient VCP occurred in 7/151 (4.6%) non-IONM patients and 24/561 (4.3%) IONM patients (*p* = 0.878), and permanent VCP occurred in 1/151 (0.7%) non-IONM patients and 2/561 (0.4%) IONM patients (*p* = 0.607). A total of 1,046 at-risk nerves were included in the analysis. Among them, transient VCP occurred in 7/246 (2.8%) nerves at-risk in the non-IONM group and 24/800 (3.0%) nerves at-risk in the IONM group (*p* = 0.901), and permanent VCP occurred in 1/246 (0.4%) nerves at-risk in the non-IONM group and 2/800 (0.3%) nerves at-risk in the IONM group (*p* = 0.688). There were no cases of bilateral VCP in either group and no significant differences in other postoperative complications between the groups.

**Table 2:** Postoperative complications in the non-intraoperative nerve monitoring and the intraoperative nerve monitoring groups

Variables	Non-IONM (*n* = 151, nerves at risk = 246)	IONM (*n* = 561, nerves at risk = 800)	*p* value
*VCP*			
Transient VCP	7 (4.6) / (2.8)*	24 (4.3) / (3.0)*	0.848 / 0.901*
Permanent VCP	1 (0.7) / (0.4)*	2 (0.4) / (0.3)*	0.607 / 0.688*
*Hypoparathyroidism*			
Transient hypoparathyroidism	5 (3.3)	35 (6.2)	0.166
Permanent hypoparathyroidism	2 (1.3)	6 (1.1)	0.792
Postoperative bleeding	0 (0.0)	6 (1.1)	0.202
Wound infection	2 (1.3)	2 (0.4)	0.158
Deep neck infection	0 (0.0)	1 (0.2)	0.604
Chyle leakage	0 (0.0)	2 (0.4)	0.462
Trachea injury	0 (0.0)	0 (0.0)	N/A

Values are expressed as *n* (%) or mean ± standard deviation

*IONM* Intraoperative nerve monitoring, *VCP* vocal cord palsy, *N/A* not applicable

*Percentage for nerves at risk

### VCP trend analysis

**Fig. 2:**
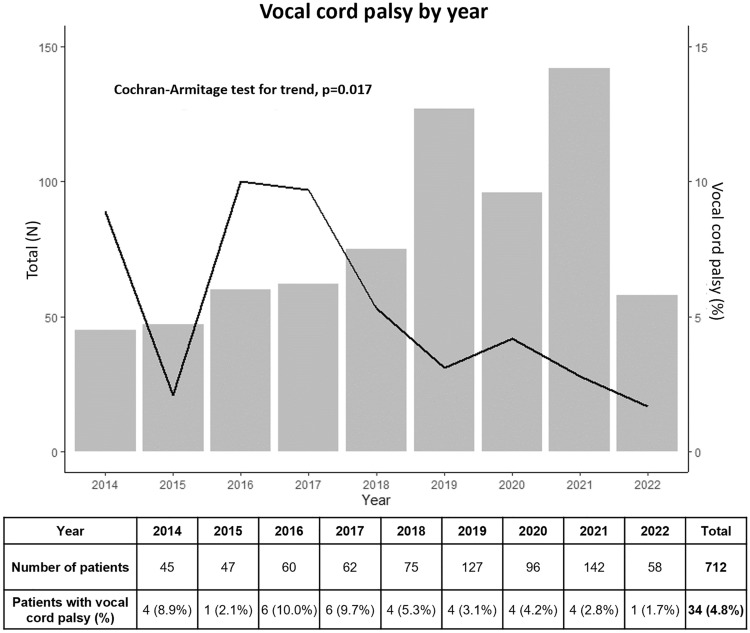
Annual vocal cord palsy rates over time. Summary of the vocal cord palsy rates (transient and permanent combined) over time. The graph shows a significant decreasing trend in vocal cord palsy rates over time as the number of cases increased (*p* = 0.017). *VCP* vocal cord palsy

Figure 2 summarizes the annual frequency and rate of VCP, pooling both transient and permanent VCP. Between 2014 and 2022, the total mean VCP rate was 4.8% and the annual VCP rates were 8.9%, 2.1%, 10.0%, 9.7%, 5.3, 3.1%, 4.2%, 2.8%, and 1.7%, respectively. As the number of cumulative cases increased over time, there was a significant decreasing trend in annual VCP rates (*p* = 0.017).

### Risk factors for vocal cord palsy

Table 3 shows the results of the risk factor analysis for VCP. In the univariate analysis, N1b stage (odds ratio [OR] 3.038, 95% confidence interval [CI] 1.285–7.182, *p* = 0.011) and extrathyroidal extension (OR 2.691, 95% CI 1.064–6.805, *p* = 0.036) were identified as significant risk factors for VCP. Four predictor variables (use of IONM, surgical extent, N stage, and extrathyroidal extension) were included in the multivariate analysis, which did not identify any significant risk factors for VCP.

**Table 3:** Risk factor analysis for vocal cord palsy

Variables	Category	*N*	E	Univariate		Multivariate	
				OR (95% CI)	*p* value	OR (95% CI)	*p* value
*Sex*	Female	485	19	Ref			
	Male	227	15	1.735 (0.865–3.481)	0.121		
Age (year)		712	34	0.988 (0.963–1.015)	0.381		
Body mass index (kg/m^2^)		707	34	1.021 (0.939–1.111)	0.626		
*Tumor location*	Right	307	12	Ref			
	Left	274	16	1.525 (0.708–3.282)	0.281		
	Bilateral	131	6	1.180 (0.433–3.214)	0.746		
*Surgical extent*	Lobectomy	378	13	Ref		Ref	
	Total thyroidectomy	334	21	1.884 (0.928–3.824)	0.080	1.328 (0.587–3.003)	0.496
*IONM use*	Non-IONM	151	8	Ref		Ref	
	IONM	561	26	0.869 (0.385–1.960)	0.735	0.886 (0.383–2.048)	0.777
*T stage*	T1	580	24	Ref			
	T2	67	6	2.279 (0.897–5.793)	0.084		
	T3 or more	65	4	1.519 (0.510–4.523)	0.453		
*N stage*	N0	420	15	Ref		Ref	
	N1a	203	10	1.399 (0.617–3.171)	0.421	1.284 (0.560–2.942)	0.555
	N1b	89	9	3.038 (1.285–7.182)	0.011	2.245 (0.832–6.060)	0.110
*Multifocality*	Single	464	24	Ref			
	Multifocal	218	10	0.942 (0.442–2.004)	0.876		
*Extrathyroidal extension*	No	656	28	Ref		Ref	
	Yes	56	6	2.691 (1.064–6.805)		1.945 (0.730–5.183)	0.183
*Thyroiditis*	No	524	22	Ref	0.036		
	Yes	188	12	1.556 (0.754–3.209)	0.232		

*N* number of patients, *E* events, *OR* odds ratio, *CI* confidence interval, *IONM* Intraoperative nerve monitoring

### Mechanism of recurrent laryngeal nerve injury

Table 4 summarizes the types of VCP and their probable mechanisms of injury. In the non-IONM group, there were seven cases of transient VCP and one case of permanent VCP, the mechanisms of injury of which were undetermined. In the IONM group, there were 24 RLN injuries causing transient VCP. They included 22 traction injuries, 1 crush injury, and 1 partial transection injury. There were also two cases of VN injury in the IONM group, with one traction injury resulting in transient VCP and other one thermal injury leading to permanent VCP.

**Table 4:** Types of vocal cord palsy and mechanisms of injury

Patients	Types of mechanism		Transient VCP (*N* = 31)	Permanent VCP (*N* = 3)
Non-IONM	Undetermined (*N* = 8)		7	1
IONM	RLN Injury (*N* = 24)	Traction injury	22	0
		Crush injury	1	0
		Thermal injury	0	1
	VN Injury (*N* = 2)	Traction injury	1	0
		Partial transection injury	0	1

*VCP* vocal cord palsy, *IONM* intraoperative neural monitoring, *RLN* recurrent laryngeal nerve, *VN* vagus nerve

## Discussion

This study was conducted to evaluate the impact of IONM on VCP rates and identify other factors that may influence the risk of VCP. We found no significant differences in VCP rates between the non-IONM and IONM groups, suggesting that the use of IONM is not associated with a lower risk of VCP. Moreover, no significant risk factors for VCP were identified by multivariate analysis. Instead, we observed a significant decrease in VCP rates over time, which may be linked to the surgeon’s cumulative experience.

One meta-analysis demonstrated that IONM minimizes the risk of VCP, and reported a significant decrease in VCP cases among IONM patients versus non-IONM patients [9]. Conversely, other meta-analyses, including those that only included randomized trials, did not find significant differences in VCP rates between IONM and non-IONM groups [19–23]. Previous studies have faced challenges in establishing the absolute superiority of IONM over non-IONM, potentially because of the small number of VCP events, particularly in surgeries performed by experienced surgeons in high-volume centers [24–26]. One study indicated that a minimum of 9,000 at-risk RLNs needs to be analyzed in randomized controlled trials to achieve sufficient statistical power to confirm the effectiveness of IONM in preventing RLN injury [25]. Another study suggested observing at least 39,907 nerves at-risk to demonstrate IONM’s superiority over non-IONM thyroid cancer surgery [24]. This study included 1,046 at-risk RLNs, which also falls short of the suggested minimum nerves at-risk. The low incidence of VCP may lead to an underestimation of the protective effect of IONM against RLN injury, and thus, the results of studies with “small” sample sizes should be interpreted with caution.

IONM may also help identify the mechanisms of RLN injuries that occur. In this study, the probable mechanism of RLN injury was able to be determined in the IONM patients, but not in the non-IONM patients. IONM can assist in characterizing RLN injury, especially in traction or compression injuries, where the RLN appears visually intact [18]. Using IONM during thyroidectomy, surgeons can modify their technique or apply other interventions to avoid repeat traction stress on the RLN and facilitate recovery. For example, if nerve function is compromised or at-risk, surgeons have options to mitigate stress on the RLN during surgery, such as administering steroids to the nerve to improve intraoperative recovery or modifying the surgical approach to the nerve [27, 28]. Although this study did not reach statistical significance on IONM’s effectiveness in preventing nerve palsy complications, we highlight its potential value as a vital tool for surgeons, including those with substantial experience, enhancing preparedness across diverse surgical scenarios.

This study showed a decrease in VCP rates over time with the increasing number of cases. This highlights the impact of surgical volume and experience on reducing the risk of VCP. Surgeons with higher case volumes and more experience are likely to possess advanced surgical skills and expertise in their field, leading to improved outcomes and reduced complications, including VCP [29]. In fact, one study demonstrated that surgeons’ experience with IONM resulted in increased RLN identification and fewer RLN injuries, even in non-IONM thyroid surgeries, indicating the value of IONM as a tool for surgical training [30]. Various studies, including both single-surgeon and multicenter studies, have also shown that a higher level of surgical experience is associated with lower rates of VCP [29, 31–33]. Studies that implemented routine IONM for thyroid surgeries reported a decline in RLN injury rates over time [29, 33]. This study confirms the ongoing reduction in recurrent nerve palsy rates as surgeons gain more experience, even after performing over 700 operations. This highlights a continual learning process in which nerve-related complications become less frequent over time, underscoring the importance of accumulating surgical skills. In real surgical settings, nerve paralysis can arise in simple as well as complex cases, such as those with variations in the recurrent laryngeal nerve or challenges during dissection, caused by thyroiditis. As surgeons accumulate experience, they become more proficient at averting nerve paralysis issues in simple or less demanding situations. Moreover, growing expertise enables surgeons to be more prepared to adeptly handle complex cases. Consistent with previous research, this study demonstrated that surgical experience using IONM (reflected by the cumulative number of cases over time) contributed to reduced rates of VCP. Further investigation into the interplay between surgical experience, the utilization of IONM, and VCP rates may augment our understanding of the factors that influence surgical outcomes in this context.

This study had several limitations. First, as a retrospective observational study, it is prone to inherent bias; however, adoption of the routine use of IONM at our center, which coincided with the national health insurance reimbursement, allowed us to minimize selection bias by stratifying patients based on the date of surgery rather than the surgeon’s preference or patient factors. The fact that patients in the IONM group underwent thyroidectomy more recently than those in the non-IONM group may have influenced the results because of the learning curve effect, which should be considered when interpreting the findings. Second, the surgical extent differed between the non-IONM and IONM groups, with significantly fewer total thyroidectomy cases than lobectomy cases in the IONM group than in the non-IONM group. This difference may be attributed to the 2015 American Thyroid Association guidelines, which expanded the indications for lobectomy to nodules up to 4 cm in size [34]. Notably, the surgical extent was not associated with the rate of VCP. Third, although this study includes 712 patients, the samples size is still insufficient to elucidate the role of IONM in reducing VCP, especially in the hands of an experienced surgeon. Finally, while this single-center, single-surgeon study may have minimized bias related to interindividual variations between surgeons, it is important to acknowledge that the data included may not be generalizable to a broader population of thyroid surgeons. Therefore, the results should be interpreted with caution considering this limitation.

In conclusion, whereas IONM may be valuable for elucidating the mechanism of injury when nerve injury occurs, the adoption of it did not decrease the rate of VCP in patients undergoing thyroid surgery for thyroid cancer. However, there was a decrease in annual VCP rates over time with an increasing cumulative number of cases, suggesting the importance of surgical volume and the surgeon’s experience for reducing VCP risk.
