# Correction: Intraoperative neuromonitoring during thyroidectomy does not decrease vocal cord palsy risk, but the cumulative experience of the surgeon may

**DOI:** 10.1007/s00595-024-02979-8

**Published:** 2024-12-28

**Authors:** Hye Lim Bae, Moon Young Oh, Mira Han, Che-Wei Wu, Young Jun Chai

**Affiliations:** 1 Department of Surgery, Seoul National University College of Medicine, Seoul National University Hospital, Seoul, Korea; 2 Department of Surgery, Seoul National University College of Medicine, Seoul Metropolitan Government Seoul National University Boramae Medical Center, Seoul, Korea; 3 Medical Research Collaborating Center, Seoul Metropolitan Government Seoul National University Boramae Medical Center, Seoul, Korea; 4 Department of Otorhinolaryngology—Head and Neck Surgery, Kaohsiung Medical University Hospital, Kaohsiung, Taiwan; 5 Transdisciplinary Department of Medicine and Advanced Technology, Seoul National University Hospital, Seoul, Korea

**Correction: Surgery Today (2024) 54:1401–1409** 10.1007/s00595-024-02871-5

In this article the author’s name Moon Young Oh was incorrectly written as: Moon Young.

The original publication has been corrected.

